# Dramatic response of metastatic cutaneous angiosarcoma to an immune checkpoint inhibitor in a patient with xeroderma pigmentosum: whole-genome sequencing aids treatment decision in end-stage disease

**DOI:** 10.1101/mcs.a004408

**Published:** 2019-10

**Authors:** Sophie Momen, Hiva Fassihi, Helen R. Davies, Christos Nikolaou, Andrea Degasperi, Catherine M. Stefanato, Joao M. L. Dias, Dhruba Dasgupta, Emma Craythorne, Robert Sarkany, Sophie Papa, Serena Nik-Zainal

**Affiliations:** 1 Department of Medical Genetics, Addenbrooke's Treatment Centre, The Clinical School, University of Cambridge, Cambridge Biomedical Campus, Cambridge CB2 0QQ, United Kingdom;; 2 National Xeroderma Pigmentosum Service, Department of Photodermatology, St John's Institute of Dermatology, Guy's and St Thomas’ Foundation Trust, London SE1 7EH, United Kingdom;; 3 MRC Cancer Unit, Hutchison/MRC Research Centre, University of Cambridge, Cambridge Biomedical Campus, Cambridge CB2 0XZ, United Kingdom;; 4 Department of Medical Oncology, Guy's and St Thomas’ NHS Foundation Trust, Great Maze Pond, London SE1 9RT, United Kingdom;; 5 Department of Dermatopathology, St John's Institute of Dermatology, Guy's and St Thomas’ Foundation Trust, London SE1 7EH, United Kingdom;; 6 Department of Nuclear Medicine, Guy's and St Thomas’ NHS Foundation Trust, Great Maze Pond, London SE1 9RT, United Kingdom;; 7 School of Cancer and Pharmaceutical Studies, King's College London, Guy's Campus, Great Maze Pond, London SE1 9RT, United Kingdom

**Keywords:** metastatic angiosarcoma

## Abstract

“Mutational signatures” are patterns of mutations that report DNA damage and subsequent repair processes that have occurred. Whole-genome sequencing (WGS) can provide additional information to standard diagnostic techniques and can identify therapeutic targets. A 32-yr-old male with xeroderma pigmentosum developed metastatic angiosarcoma that was unresponsive to three lines of conventional sarcoma therapies. WGS was performed on his primary cancer revealing a hypermutated tumor, including clonal ultraviolet radiation-induced mutational patterns (Signature 7) and subclonal signatures of mutated DNA polymerase epsilon (*POLE*) (Signature 10). These signatures are associated with response to immune checkpoint blockade. Immunohistochemistry confirmed high PD-L1 expression in metastatic deposits. The anti-PD-1 monoclonal antibody pembrolizumab was commenced off-label given the *POLE* mutation and high mutational load. After four cycles, there was a significant reduction in his disease with almost complete resolution of the metastatic deposits. This case highlights the importance of WGS in the analysis, interpretation, and treatment of cancers. We anticipate that as WGS becomes integral to the cancer diagnostic pathway, treatments will be stratified to the individual based on their unique genomic and/or transcriptomic profile, enhancing classical approaches of histologically driven treatment decisions.

## INTRODUCTION

Xeroderma pigmentosum (XP) is a rare, autosomal recessive disorder of nucleotide excision repair (NER), with an incidence in Western Europe of 2–3 per million live births (Kleijer et al. 2008). There are eight different complementation groups, dependent on the XP protein that is mutated (XP-A through G and V). The XP-A to G proteins are involved in repairing bulky DNA damage caused by ultraviolet radiation (UVR)—namely, cyclobutane pyrimidine dimers (CPDs) and 6–4 pyrimidine-pyrimidone photoproducts (Brash 2015). Unrepaired CPDs cause a UVR-related mutational signature characterized by C > T and CC > TT substitutions (Brash 2015). As a result of the excess of UVR-related damage, patients develop exposed-site pigmentary changes, multiple skin cancers, and ocular surface disease and one-third of patients develop progressive neurodegeneration (Fassihi et al. 2016). XP patients have a 2000-fold increased incidence of melanoma, and a 10,000-fold increased incidence of non-melanoma skin cancer (Bradford et al. 2011).

The immune system plays a critical role in the surveillance of malignant cells. However, its effectiveness can be restricted by various immune-escape or checkpoint mechanisms. In particular, the programmed death protein (PD-1) binds to ligands PD-L1 and PD-L2. Upon activation, T cells express PD-1, which interacts with PD-L1 on tumor and stromal cells, deactivating the T cells and negatively regulating T-cell effector functions (Gentzler et al. 2016).

Immune checkpoint inhibitors interfere with these checkpoints, reactivating antitumor activity of cytotoxic T cells. Immunotherapies like anti-PD-1 and anti-PD-L1 have revolutionized the treatment of certain malignancies, especially melanoma (Gentzler et al. 2016). Their role in treating other solid organ malignancies is expanding (Gentzler et al. 2016). Higher tumor PD-L1 levels may be an indicator of an enhanced response to treatment. However, the role of PD-L1 as a predictive biomarker is controversial, as there is a subset of PD-L1 negative tumors that still respond to PD-1/PD-L1 inhibition (Gentzler et al. 2016). Tumors with high mutational loads have been shown to demonstrate a greater response to immune checkpoint blockade (Howitt et al. 2015). One explanation for this is the creation of a large number of neoantigens. Nevertheless, other factors are likely to play a role, as not all hypermutated tumors respond in the same way (Howitt et al. 2015).

Somatic mutations in the proofreading exonuclease domain of DNA polymerase epsilon (*POLE)* have been associated with tumors of high mutational loads (Shinbrot et al. 2014). Mutations in *POLE* have been reported in colorectal and 10% of endometrial cancers (Gargiulo et al. 2016). In a study of hypermutated endometrial cancers (>232 × 10^−6^ mutations/Mb), tumors with somatic *POLE* mutations were associated with a 15-fold higher number of neoepitopes per sample when compared to tumors with microsatellite instability (MSI). *POLE*-mutated and MSI tumors also had a higher number of CD3^+^ (*P* = 0.001) and CD8^+^ (*P* < 0.001) tumor-infiltrating lymphocytes (TILs) compared to microsatellite-stable tumors. *POLE* mutations are associated with increased PD-1 and PD-L1 expression (Howitt et al. 2015). PD-1 was shown to be overexpressed in TILs and peritumoral lymphocytes of *POLE*-mutated tumors, thus suggesting that *POLE*-mutated tumors may be candidates for PD-1/PD-L1-targeted immunotherapies (Howitt et al. 2015). *POLE* mutations have been demonstrated in an extensive number of tumor types that have shown sensitivity to checkpoint inhibitors (Gargiulo et al. 2016).

## RESULTS

### Case

**Figure 1.**
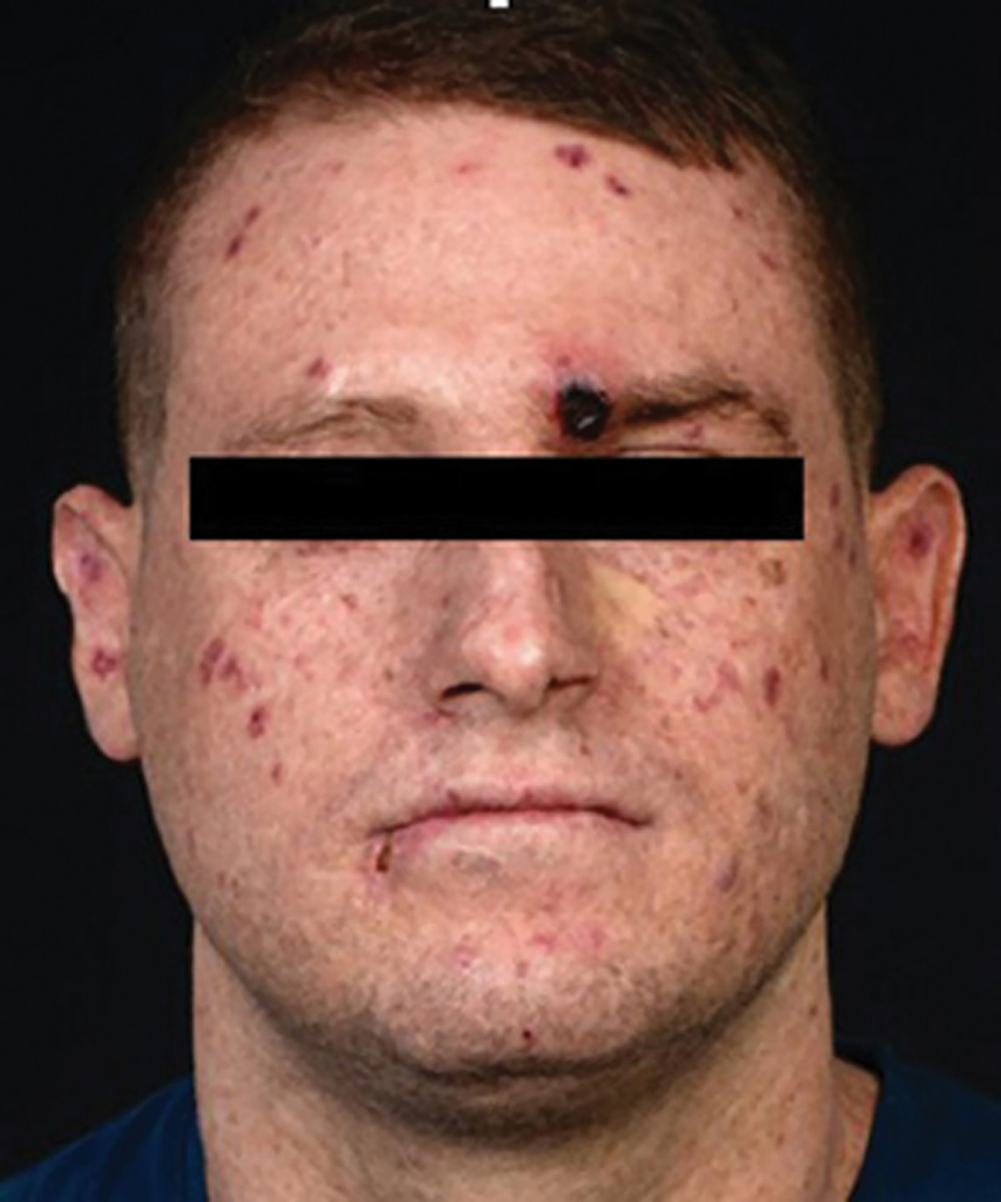
Clinical image of patient highlighting angiosarcoma on the medial aspect of his left eyebrow.

**Figure 2.**
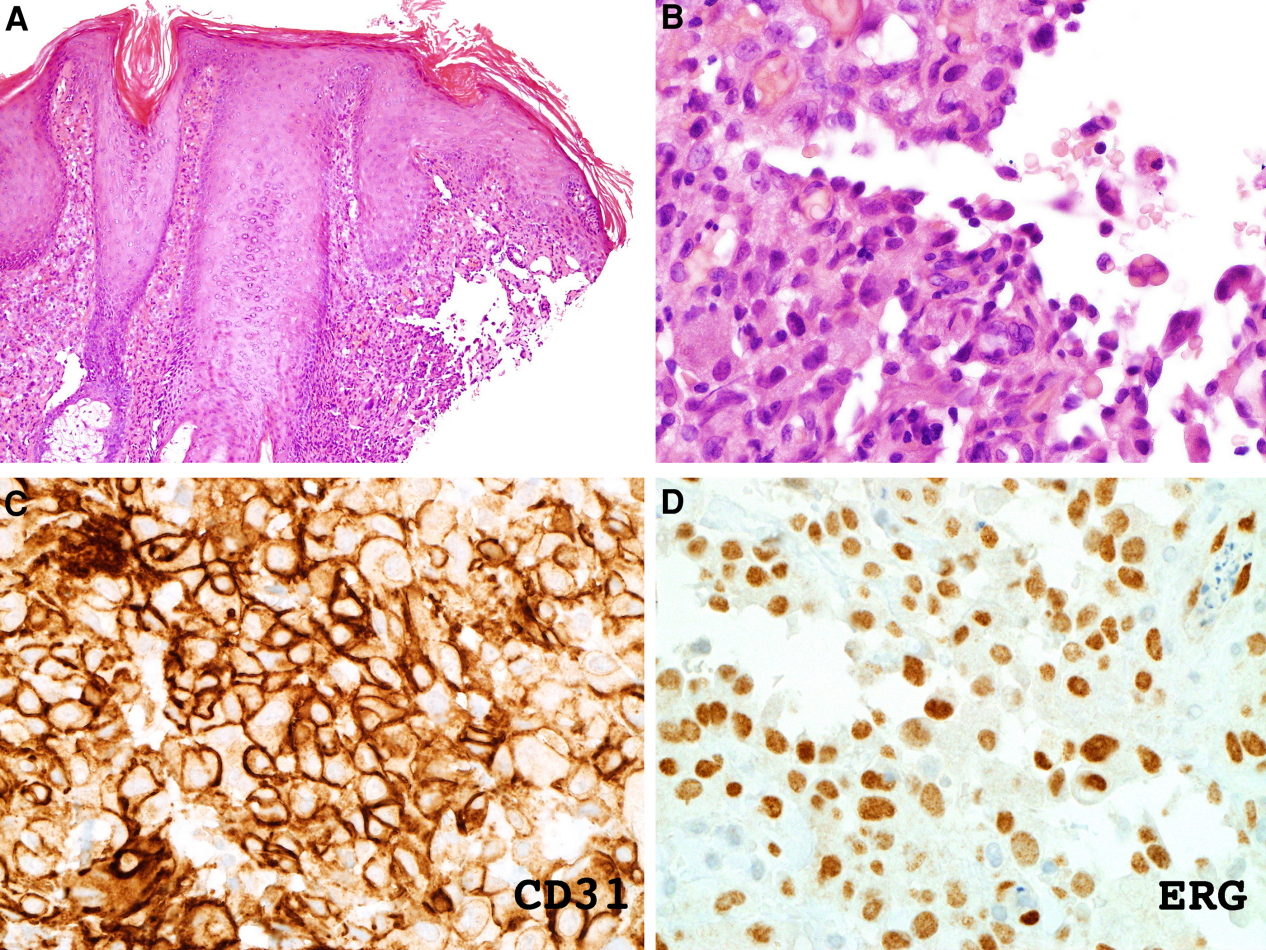
(*A*,*B*) Hematoxylin and eosin staining of skin biopsy shows dermal infiltrate of pale eosinophilic cells that have characteristic features of “dissecting the collagen bundles” and atypical, enlarged, and hyperchromatic nuclei. (*C*,*D*) Immunostaining shows staining for vascular markers CD31 and ERG (labeled).

**Figure 3.**
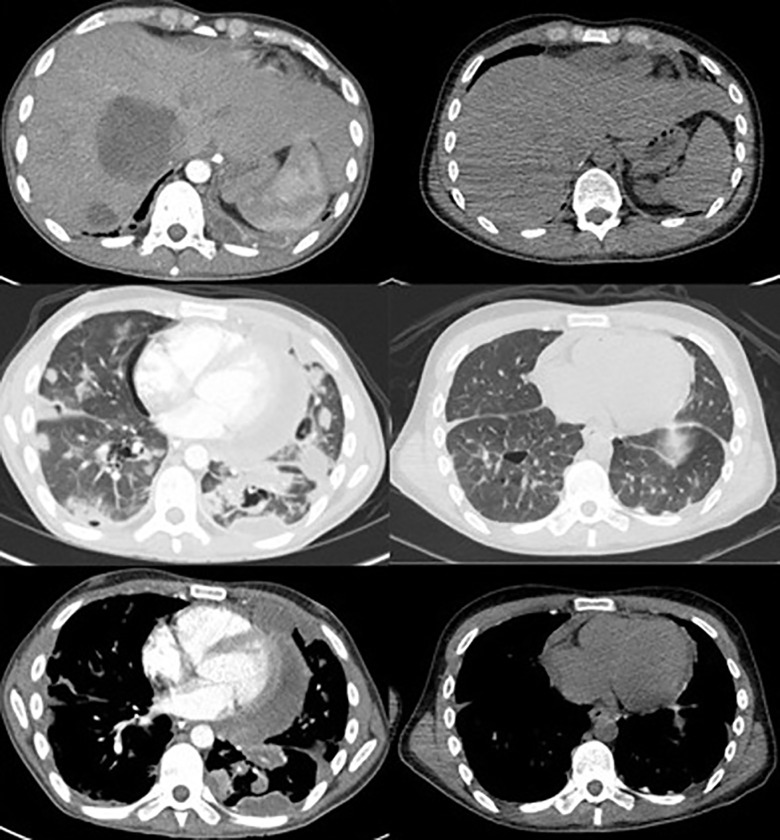
Figure showing response of disease after four cycles of pembrolizumab. Pretreatment CT image (*left*) and PET-CT scan after four cycles of pembrolizumab (*right*). The *top* row shows a reduction in the size of liver metastases; the *middle* row shows a reduction in the size of lung metastatic deposits; and the *bottom* row shows the mediastinal response.

**Figure 4.**
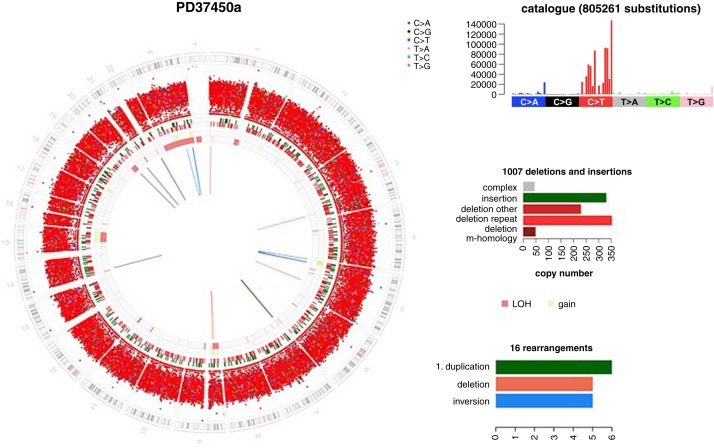
Circos plot of whole-genome sequenced angiosarcoma. It depicts a chromosomal ideogram on the outermost ring. Moving inward, the next ring shows a large number of C > T transitions. The next ring depicts small (<100-bp) insertions (green) and deletions (red). Then the next rings report copy-number state (green = gains, pink = losses), and the lines in the center of the plot report structural variants, of which there are not many. The *right-hand top* panel displays the substitution mutation profile. This graph shows that there are 805,261 C > T transitions with a mutational profile that is typical of UV damage. The *right-hand middle* panel shows the distribution of classes of indels, of which there are 1007. The *right-hand bottom* panel shows the types of structural variants that are present in this tumor.

**Figure 5.**
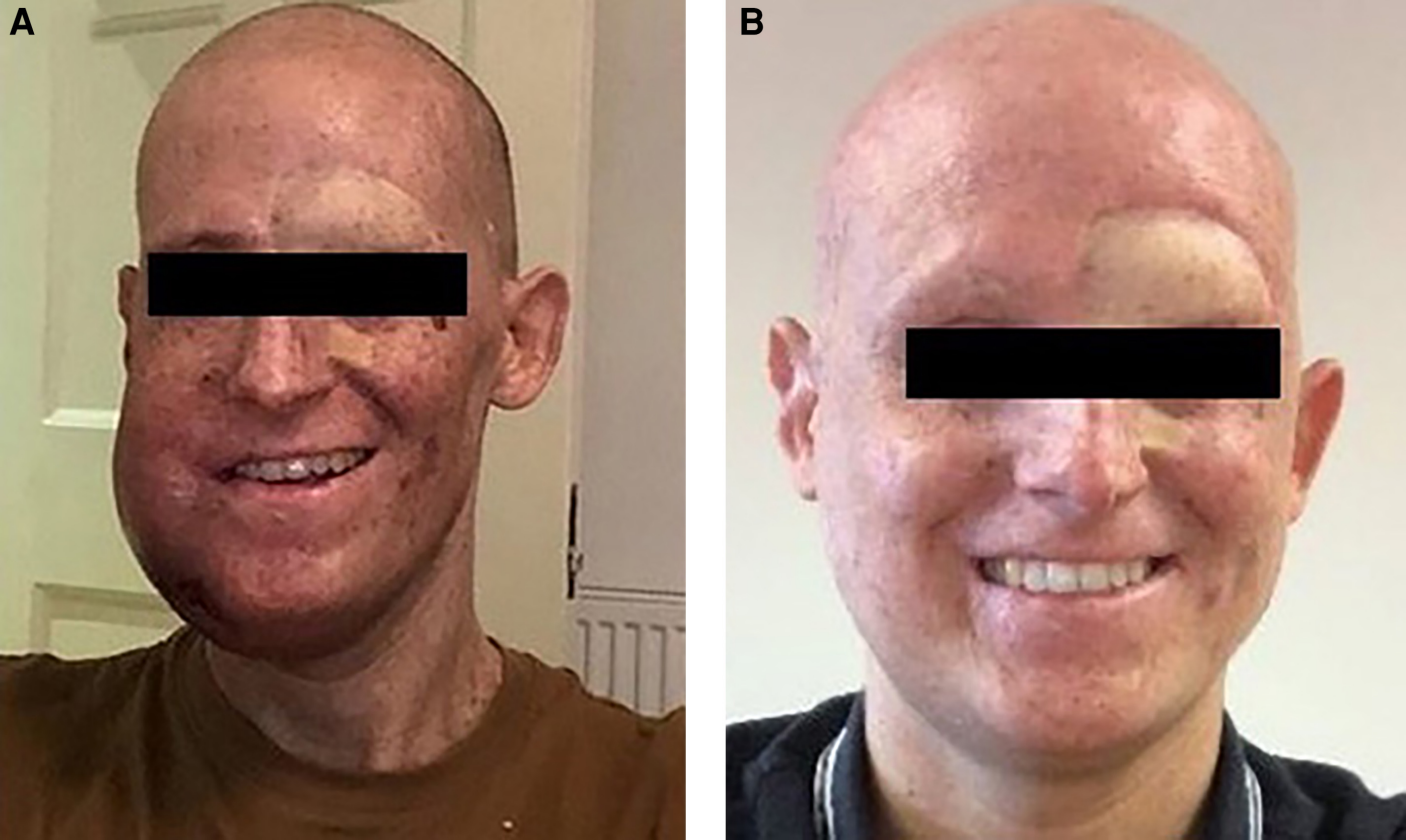
Clinical images showing large soft tissue mass on left jaw that was causing tracheal compression (*A*) and post three cycles of pembrolizumab (*B*).

A 32-yr-old Caucasian male with XP complementation group C (compound heterozygous mutations; c.445_446delGA in exon 4a and c.2336 del T in exon 13 in *XPC*) (XP1SH) presented in January 2017 with a rapidly growing violaceous nodule on the left supraorbital area (Fig. 1). He had a history of multiple non-melanoma skin cancers. Histopathology demonstrated a dense dermal infiltrate of pale, eosinophilic, neoplastic cells extending from the papillary to the reticular dermis. These cells “dissected the collagen bundles” and had enlarged and hyperchromatic nuclei (Fig. 2A,B). Immunostaining was positive for vascular markers CD31 and ERG (Fig. 2C,D). The neoplastic cells were negative for S100, CD34, and MFT 116. All these findings were in keeping with the diagnosis of cutaneous angiosarcoma. Positron emission tomography–computed tomography (PET-CT) showed no metastatic disease. A wide local excision was undertaken at the regional sarcoma unit, and clear margins were reported. Adjuvant radiotherapy was not given. Eight months later, he presented with a right submandibular palpable mass; fine-needle aspiration confirmed metastatic angiosarcoma. A repeat PET-CT showed bilateral submandibular nodes. He was commenced on ifosfamide and doxorubicin at a standard dosing, which was well-tolerated. After six cycles, a PET-CT showed metastatic disease in the lung and liver. Eighteen months after diagnosis he was enrolled into the TAPPAS trial of TRC105 (carotuximab, a monoclonal antibody targeting CD105) and pazopanib versus pazopanib alone. After 1 month there was suspicion of clinical disease progression, and upon repeat imaging, further metastatic disease was confirmed, with mediastinal, pericardial, pleural, liver, and bony infiltration (Fig. 3). A base-of-skull metastasis demonstrated direct cranial extension. His Eastern Collaborative Oncology Group-Performance Status (ECOG-PS) was 3 and he was admitted as an inpatient with severe cachexia, anorexia, and concerns about imminent terminal complications of his disease. Bilateral pneumothoraces were treated with video-assisted thoracic surgery (VATS) procedures and pleurodeses. Palliative radiotherapy 36 Gy in 12 fractions was given to a mandibular tumor deposit because of impending tracheal compression (Fig. 4). WGS was performed on a primary tumor biopsy (Fig. 5).

### Genomic Analysis

Driver mutations were identified in *TP53* (H179Y, variant allele fraction of 26%) and *CDKN2A* (homozygous whole-gene deletion). Driver mutations linked to angiogenesis previously reported in angiosarcomas, *PTPRB* and *PLCG1*, were not observed (Behjati et al. 2014). Mutations in *TP53* and homozygous deletions of *CDKN2A* have previously been reported in angiosarcomas (Behjati et al. 2014). WGS additionally revealed an extremely high mutational load of 805,261 substitutions and 1007 small insertions/deletions (indels) (Fig. 5). Two base substitution mutational signatures were identified. Signature 7, the classic “UV signature” (Alexandrov et al. 2013) comprising C > T transitions and CC > TT double substitutions accounted for 91.2% of these. This is the mutational signature that is commonly seen in melanoma (Pleasance et al. 2010). In a patient with deficient NER, this signature would be expected, even in noncancerous “normal” skin. However, Signature 10 was also identified, accounting for a small fraction of 8.8% of the mutational load in the primary tumor. This signature is characterized by three distinctive substitution peaks—C > A substitutions at TCT trinucleotides, C > T substitutions at TCG trinucleotides and T > G substitutions at TTT trinucleotides—and is reported in association with activating driver mutations in the proofreading exonuclease domain of *POLE* (Cancer Genome Atlas Network 2012). The possibility that this mutation was present in a subclone of the primary tumor was raised. Closer inspection revealed a subclonal driver mutation in *POLE* (S459F, variant allele fraction of 17%). Although it is a noncanonical *POLE* mutation, it maps to the Exo III motif of the endonuclease. Functional work supports reduced proofreading activity of an expression construct carrying this mutation, supporting driver potential, and it is this mutation that was driving the patient's metastatic disease (Shinbrot et al. 2014).

**Figure 6.**
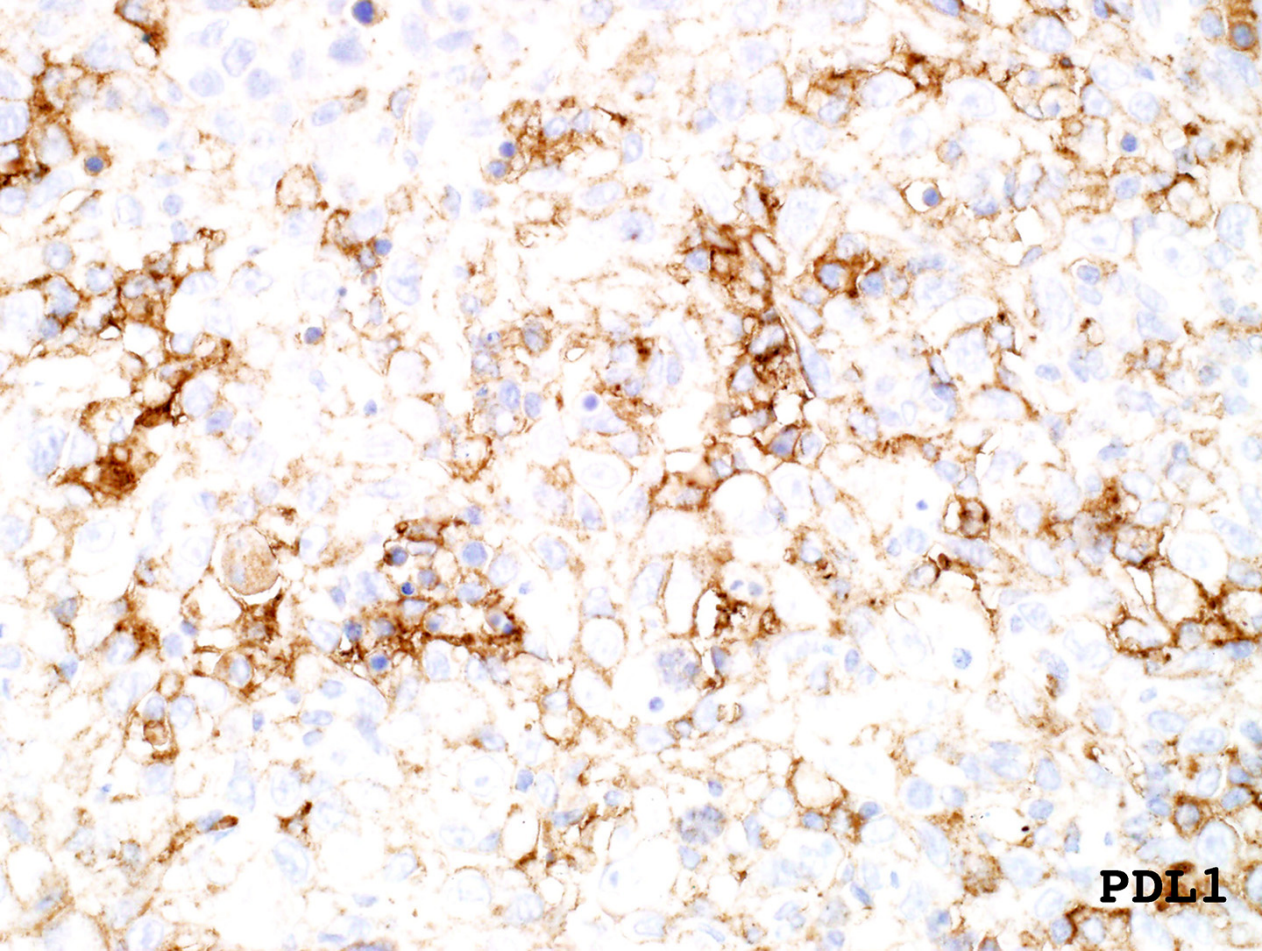
Immunohistochemistry performed on primary excision staining positive for PD-L1 (60%).

Both a high mutational burden and somatic mutations in *POLE* are reported to be predictors of sensitivity to immune checkpoint inhibitors. Consistent with this prediction, immunohistochemistry of PD-L1 was positive on both the primary excision and on a metastatic lymph node deposit (60% positivity) (Fig. 6). However, anti-PD-/PD-L1 therapy for angiosarcoma is not currently licensed given the lack of clinical trial data supporting its use (Table 1).

**Table 1.** Genomic analysis of the patient's primary angiosarcoma

Gene	Chromosome	Genomic location	HGVS DNA reference	HGVS protein reference	Variant type	Predicted effect	dbSNP/dbVar ID	Genotype
*POLE*	12	g.133249847G > A	c.1376C > T	p.S459F	Sub	Missense		Heterozygous
*TP53*	17	g.7578395 G > A	c.535C > T	p.H179Y	Sub	Missense	rs587780070	Heterozygous
*CDKN2A*	9	g. 21694602–23444942			Homozygous deletion	Whole gene deletion		Homozygous

### Treatment Outcomes

The anti-PD-1 monoclonal antibody pembrolizumab at 200 mg every 3 weeks was commenced off-label given the *POLE* mutation and high mutational load. Treatment was tolerated well with no side effects. After four cycles there was evidence of resolution of the lung and bone disease and almost complete resolution of the cardiac disease and pericardial effusions. There was significant reduction in the liver metastases and the residual visible liver disease was no longer FDG-avid (Fig. 3). Activity remained in a soft tissue jaw mass and pleural tissue; however, these were significantly reduced in volume (Fig. 4). The remaining jaw uptake is suggestive of osteoradionecrosis and may not represent ongoing malignant disease. His weight has increased from 48 kg to 56 kg and ECOG-PS is 0. Diuretics, high dose steroids, and opiates have been weaned completely. He has now had 11 cycles, he is back at work full-time, and treatment is ongoing.

## DISCUSSION

### Angiosarcoma and XP

Cutaneous angiosarcomas are rare malignancies of endothelial cells that often occur on the head and neck of the elderly (Griffiths et al. 1998). They arise spontaneously or secondary to ionizing radiation or chronic lymphoedema (Griffiths et al. 1998). Angiosarcoma has a poor prognosis, and metastatic disease is common. It has an estimated mean 5-yr survival rate of 33.5% (Griffiths et al. 1998). Genomic studies of angiosarcomas have identified key driver mutations in genes linked to angiogenesis (Behjati et al. 2014). Truncating mutations in *PTPRB* (an endothelial phosphatase and negative regulator of vascular growth factor tyrosine kinases) and activating Arg707Gln missense mutations in *PLCG1* (a signal transducer of tyrosine kinase) have been reported (Behjati et al. 2014).

These mutations were not present in our case. Angiosarcoma is rarely seen in the context of XP; there are 11 reports of angiosarcoma in XP patients, with most of these (*n* = 10) occurring on the head and neck (Leake et al. 1992; De Silva et al. 1999; Ludolph-Hauser et al. 2000; Marcon et al. 2004; Arora et al. 2008; Olson et al. 2012; Sharma et al. 2012; Karkouche et al. 2013). Of these, seven patients were treated with surgical excision, three with surgery plus radiotherapy, and one with chemotherapy. Only two patients had local recurrence, and no metastatic disease was observed over a mean follow-up period of 1–40 months. Chemotherapy and radiotherapy can be used in patients with XP in most cases, but specialist advice is suggested. Anti-PD-L1 inhibition has been successfully used for the treatment of metastatic melanoma and metastatic cutaneous squamous cell carcinomas in patients with XP (Deinlein et al. 2017; Salomon et al. 2018).

### Angiosarcoma and Its Treatment

Cutaneous angiosarcoma is managed with surgical excision of the primary tumor, with or without postoperative radiotherapy. Chemotherapy with doxorubicin or paclitaxel has been used for metastatic disease, and based on data from retrospective and prospective trials, its true value is unclear. Sindhu et al. (2017) report a patient with metastatic angiosarcoma expressing PD-L1, treated with off-label pembrolizumab 2 mg/kg every 21 d for 13 cycles with shrinkage of his liver disease and no new facial lesions during an 8-mo follow-up period off treatment. To date, there are no immunotherapy agents licensed to treat angiosarcoma. Furthermore, secondary autoimmune-related complications have been reported with checkpoint inhibitor therapy. This is the first report of a patient with end-stage metastatic angiosarcoma on the background of XP being treated with immunotherapy.

## CONCLUSION

In the last decade, whole-genome sequencing has enabled us to visualize every mutation present in a human cancer genome, allowing us to gain insights into the drivers and mutational signatures present in an individual's cancer. Mutational signatures can offer unique insights into the pathogenesis of a cancer and provide information that may be translated into possible therapeutic and preventative treatments (Nik-Zainal et al. 2016). In this case, mutational signatures from WGS have been used as a biomarker to guide immunotherapy. Our patient had none of the classical “drivers” seen in an angiosarcoma, possibly explaining why he progressed after three lines of “standard” treatment. The histological appearances were a poor predictor of treatment response, whereas the molecular fingerprint was an excellent predictor of response to therapy with pembrolizumab. We hope that as WGS becomes an integral part of cancer care, cancer treatments will be stratified to the individual based on their unique mutational makeup as opposed to the classical method of histological based treatment decisions. Because formal clinical trials are not possible in ultra-rare situations such as this one, mutational fingerprinting in individual cases and small patient cohorts will be critical in providing effective therapies to these patients.

## Methods

### Immunohistochemistry

Immunohistochemistry was performed and positive for the vascular markers CD31 and ERG. Negative markers included S100, CD34, and MFT 116.

### Genomic sequencing

WGS was performed on genomic DNA from the original tumor and peripheral blood lymphocyes, using Illumina HiSeq X TEN, 150-bp paired-end sequencing technology. Average tumor coverage was 35.6× and average normal coverage was 31.09× (Table 2). Short reads were aligned to build GRCh37 using BWA v2.0.54, substitutions called using CaVEMan v1.11.2, indels called using Pindel v2.2.2, structural variation called using Brass v5.4.1, and copy number using ASCAT (NGS) v4.0.1. Total ploidy was estimated to be 1.85.

**Table 2.** Table showing quality control metrics for whole-genome sequencing

Sequence metrics	Sequencing method	Whole-genome sequencing
	Tumor coverage	35.6×
	Normal coverage	31.09×
	Additional text	Duplicate read rate < 10%
Processing	Alignment	BWA v2.0.54
	Substitutions	CaVEMan v1.11.2
	Indels	Pindel v2.2.2
	Rearrangements	Brass v5.4.1
	Copy number	ASCAT (NGS) v4.0.1
Raw data	Substitutions	805,261
	Indels	1007
	Rearrangements	11
Ploidy	1.85	
Aberrant cell fraction	0.35	

## ADDITIONAL INFORMATION

### Data Deposition and Access

The raw sequencing data has been submitted to the European Genome-phenome Archive (EGA) (https://ega-archive.org/) under accession number EGAD00001004786. The variants have been uploaded to Mendeley and can be found under doi number: 10.17632/7cxt72pckw.1 (https://data.mendeley.com/datasets/7cxt72pckw/1).

### Ethics Statement

The patient has consented for all aspects of his care to be published including clinical photography. WGS was performed as a part of a clinical research project and was approved by the U.K. National Research Ethics Service.

### Acknowledgments

We thank Professor Alan Lehmann, University of Sussex, for an expert opinion on DNA repair and xeroderma pigmentosum, and Dr Robin Jones, Royal Marsden Hospital, London, for delivery of the clinical trial.

### Funding

This work was funded by a Cancer Research UK (CRUK) Advanced Clinician Scientist Fellowship (C60100/A23916) and a Wellcome-Beit Prize.

### Competing Interest Statement

S.N.-Z. is an inventor on four published patents and one additional patent application in review, none of which is relevant to the current manuscript.
